# Cognitive Decline in Chronic Coronary Syndrome: Associations with Vascular, Cardiac, and Neuropsychological Parameters

**DOI:** 10.3390/medicina62071239

**Published:** 2026-06-26

**Authors:** Marius Militaru, Daniel Florin Lighezan, Florina Buleu, Stela Iurciuc, Daian-Ionel Popa, Anda Gabriela Militaru

**Affiliations:** 1 Department of Neuroscience, University Clinic of Neurology II, “Victor Babes” University of Medicine and Pharmacy, E. Murgu Square, Nr. 2, 300041 Timisoara, Romania; marius.militaru@umft.ro; 2 Emergency City Hospital Timisoara, Gheorghe Dima Street Nr. 5, 300254 Timisoara, Romania; dlighezan@umft.ro (D.F.L.); daian-ionel.popa@umft.ro (D.-I.P.); militaru.anda@umft.ro (A.G.M.); 3 Centre of Advanced Research in Cardiology and Hemostasology, “Victor Babes” University of Medicine and Pharmacy, E. Murgu Square, Nr. 2, 300041 Timisoara, Romania; 4 Department of Internal Medicine I, University Clinic of Medical Semiology I, “Victor Babes” University of Medicine and Pharmacy, E. Murgu Square, Nr. 2, 300041 Timisoara, Romania; 5 Department of Cardiology, “Victor Babes” University of Medicine and Pharmacy, E. Murgu Square, Nr. 2, 300041 Timisoara, Romania; 6 Research Center for Medical Communication, “Victor Babes” University of Medicine and Pharmacy, 300041 Timisoara, Romania

**Keywords:** chronic coronary syndrome, cognitive decline, severe cognitive impairment, BMI, depression, neuropsychological assessment

## Abstract

*Background and Objectives:* A relationship between cognitive decline (CD) and chronic coronary syndrome (CCS), common among the elderly population, has not yet been clearly established. Our study aims to evaluate the link between severe cognitive impairment and cognitive impairment, as measured by various neuropsychological tests in patients with or without CCS. In addition, we sought to identify cardiovascular risk factors (CVRFs) that influence the severity of CD and severe cognitive impairment. *Materials and Methods:* This observational study was conducted on 264 people with CVRFs. Of the 264, 132 were classified as patients with CCS and 132 as control subjects without CCS. Neuropsychological assessment tools included the Instrumental Activities of Daily Living (IADL) and Activities of Daily Living (ADL) scales, the Montreal Cognitive Assessment (MoCA), the Mini-Mental State Examination (MMSE), and the Geriatric Depression Scale (GDS-15). Clinical characteristics, echocardiographic measures, and vascular parameters of all subjects were also evaluated. *Results:* Patients with CCS had significantly lower cognitive performance (MMSE, *p* = 0.010; MoCA, *p* = 0.021), reduced functional status (IADL, *p* = 0.030; ADL, *p* = 0.012), and higher depression scores (*p* = 0.004) compared with controls. They also had worse cardiovascular profiles, including lower left ventricular ejection fraction (LVEF) (*p* = 0.001), higher NT-proBNP levels (*p* = 0.005), and increased carotid intima-media thickness (IMT) (*p* < 0.05). IMT and blood pressure values were negatively correlated with cognitive and functional scores and positively correlated with depression severity (*p* < 0.001). Multivariate analysis identified systolic and diastolic blood pressure, age, body mass index, heart rate, reduced daily activity, and depression as independent predictors of cognitive decline in patients with CCS. In the GDS-15 score, each unit increase was associated with a 32.1% higher risk of cognitive decline and a 37.1% higher risk of MMSE-defined severe cognitive impairment, while improved ADL scores significantly reduced this risk. *Conclusions:* CCS is associated with an increased risk of severe cognitive impairment and also with cognitive decline, influenced by hypertension, subclinical atherosclerosis, depression, and reduced functional status. These findings emphasize the importance of early identification and multidisciplinary management of cognitive impairment in patients with CCS to prevent progression to severe cognitive impairment.

## 1. Introduction

Chronic coronary syndrome (CCS) represents the stable expression of coronary artery disease and is responsible for a significant portion of cardiovascular disease and death around the world. The chronic coronary syndrome concept was first introduced by the European Society of Cardiology in 2019 [1] and further defined in the 2024 ESC Guidelines as a set of clinical syndromes resulting from structural and/or functional abnormalities of the coronary arteries and/or coronary microcirculation [2]. This concept encompasses patients with both obstructive and non-obstructive coronary artery disease, including angina or ischemia with non-obstructive coronary arteries (ANOCA/INOCA), as well as conditions that occur after revascularization or are related to microvascular or vasospastic mechanisms [2,3]. Epidemiological evidence suggests that an unfavorable CVD risk profile significantly increases the likelihood of developing cognitive impairment [4]. Cardiovascular disease and cognitive impairment frequently coexist, creating a substantial burden for patients, caregivers, and healthcare systems [5,6].

Several mechanisms could explain the association between CCS and cognitive decline. Common cardiovascular risk factors of advanced age, hypertension, diabetes mellitus, dyslipidemia, obesity, smoking, and atrial fibrillation are shared by both conditions [4,7]. Vascular abnormalities such as carotid and intracranial atherosclerosis might lead to cerebral hypoperfusion, endothelial dysfunction, and ischemic brain injury, accelerating cognitive decline [8]. Impaired cardiac function may also negatively impact cerebral perfusion. Previous studies have demonstrated that even in the absence of overt heart failure, reduced left ventricular ejection fraction (LVEF) is related to worse cognitive performance [9]. Recent evidence lends support to the heart-brain axis; cardiovascular dysfunction may contribute directly to brain injury and neurodegeneration [10].

Increasing evidence links coronary heart disease with an increased risk of cognitive impairment and severe cognitive impairment. Cognitive impairment affects about one-third of cardiovascular disease patients and is often unnoticed in regular clinical practice [6]. Two meta-analyses have shown coronary heart disease to be related to approximately a 26–45% increased risk for future cognitive decline or severe cognitive impairment [11,12]. Importantly, 1/3 of all severe cognitive impairment cases can be linked back to modifiable CVRFs, indicating the opportunity for prevention with targeted intervention [6,13].

As much as one-third of all severe cognitive impairment cases may be linked to potentially modifiable cardiovascular risk factors. It emphasizes how important it is to try to stop severe cognitive impairment by preventing vascular problems [6,9]. Despite these findings, cognitive assessment remains insufficiently integrated into routine cardiovascular care, and relatively few studies have evaluated CCS as a distinct clinical entity using a comprehensive approach that incorporates vascular, cardiac, functional, and psychological parameters [6,14].

Hence, we set out to assess whether CCS is related to cognitive deterioration in subjects with cardiovascular risk factors. We studied cognitive function, functional status, depressive symptoms, vascular characteristics, and echocardiographic parameters in those with and without CCS. We hypothesized that patients with CCS would perform worse on cognitive and functional measures and that cardiovascular, vascular, and psychological factors would independently relate to cognitive impairment. Such information would help in the early detection and multidisciplinary treatment of cognitive dysfunction in patients with CCS.

## 2. Materials and Methods

### 2.1. Study Population and Design

In this observational study, we examined a sample of 264 consecutive hospitalized patients admitted between 1 June 2021 and 15 December 2024 to the Internal Medicine Department of the Municipal Emergency Hospital of Timișoara, Romania, who met the inclusion criteria to assess neurologic symptoms and/or evaluate previously diagnosed chronic coronary syndrome. All patients had CVRFs and reported progressive memory impairment, suggestive of cognitive decline, without a prior diagnosis of severe cognitive impairment or CD and without specific treatment for these conditions. Patients with CCS were clinically stable and were receiving optimal drug therapy.

Patients were grouped by CCS status: group A included 132 patients with CCS and CVRFs, while group B included 132 control subjects with CVRFs but without CCS.

The inclusion criteria for participants were individuals aged 40 or older who were hospitalized during this research project. All subjects had to have at least two and no more than four of the following risk factors for cardiovascular disease: high blood pressure, diabetes, dyslipidemia, smoking, obesity, and a family history of premature cardiovascular disease. In addition, all subjects had to meet clinical criteria for suspected cognitive impairment (i.e., having experienced progressive memory loss or exhibited neurocognitive symptoms) and have no prior diagnosis of cognitive impairment or severe cognitive impairment. Patients with a documented diagnosis of CCS were included in the study group of symptomatic patients with CCS, while patients without CCS but with comparable cardiovascular risks were included in the control group. Finally, all participants had to be clinically stable and able to undergo a comprehensive cardiovascular and neuropsychological evaluation. To minimize the inclusion of individuals at low risk or with multiple other health problems, and to avoid confounding the results regarding the relationship between CVRFs, CCS, and cognitive outcomes in this study, each included subject had to have a cardiovascular risk profile similar to that of the general population.

Exclusion criteria were myocardial infarction, new-onset or decompensated heart failure, signs or symptoms of acute coronary syndrome, uncontrolled hypertension, severe chronic diseases that would seriously limit the subject’s ability to participate in the study, psychiatric illnesses, or a previous diagnosis of severe cognitive impairment. Patients under treatment for severe cognitive impairment were also excluded. Also, the study did not include separate groups for patients with angina and non-obstructive coronary arteries (ANOCA) or ischemia with non-obstructive coronary arteries (INOCA); these cohorts were also not specifically assessed in the current analysis.

All participants completed a comprehensive clinical, cardiological, and neurological evaluation. The clinical evaluation included medical history, physical examination, heart rate (HR), and systolic and diastolic blood pressure (SBP and DBP). Blood tests included lipid profile, renal and hepatic function parameters, N-terminal B-type natriuretic peptide (NT-proBNP), and the triglyceride-glucose index (TyG). CCS diagnosis and classification were made in line with the guidelines of the European Society of Cardiology [2].

All patients underwent resting ECG, ankle-brachial index (ABI) measurement, carotid Doppler ultrasound to assess intima-media thickness (IMT), and transthoracic echocardiography (TTE). A mandatory brain CT scan was not required for the diagnosis of BC.

Validated tools were used to assess cognitive and functional status, including the Mini-Mental State Examination, the Montreal Cognitive Assessment, Activities of Daily Living, Instrumental Activities of Daily Living, and the Geriatric Depression Scale [15,16,17,18,19,20,21,22].

This study followed the Declaration of Helsinki, and it was approved by the Ethics Committee of the Municipal Emergency Hospital in Timișoara, Romania (No. E1518/17 March 2021). All participants signed an informed consent form.

### 2.2. Clinical and Cognitive Assessments

#### 2.2.1. Cardiac Assessment

The echocardiogram was acquired using a General Electric Vivid F9 system (GE Vingmed Ultrasound AS, Norway), equipped with an M5S transducer, in accordance with current guidelines [2].

Two-dimensional echocardiography was used to assess cardiac chamber dimensions and function. Left ventricular ejection fraction was calculated using the Simpson biplane method. Diastolic function was assessed using transmitral Doppler flow (where E and A are the E/A ratio). Myocardial deformation was assessed using speckle-tracking echocardiography to determine global longitudinal deformation. Tissue Doppler imaging (TDI) was used to measure E′, A′, S′ velocities, E′/A′ ratio, and mitral annular plane systolic excursion (MAPSE). Additional parameters, including valvular function and pericardial assessment, were also recorded [14].

The examination was performed in standard apical views (2-, 3-, and 4-chamber views) to assess the size and function of the left ventricle (LV) and left atrium (LA). All Global longitudinal strain (GLS) measurements were performed using speckle-tracking imaging [14]. All imaging examinations were performed by the same qualified examiner using the same ultrasound machine and using standardized protocols.

#### 2.2.2. Vascular Assessment

Carotid intima-media thickness was assessed using Doppler ultrasound (GE Vivid E9 system with a 9 MHz transducer). Measurements were taken at the level of the common carotid artery, 1 cm proximal to the carotid bulb. Ten measurements were taken for each patient, and the mean value was used for analysis [23].

The ankle-brachial index was calculated as the ratio of systolic blood pressure measured at the ankle to that measured at the arm. Values < 0.90 were considered indicative of peripheral arterial disease, 0.91–0.99 borderline, and 1.0–1.4 normal [24].

#### 2.2.3. Metabolic Assessment

The triglyceride–glucose index was calculated as ln [fasting triglycerides (mg/dL) × fasting plasma glucose (mg/dL)/2] and used as a surrogate marker of insulin resistance. Fasting blood samples were obtained after an overnight fast of at least 8–12 h, and biochemical parameters were analyzed using standard laboratory methods [25].

The TyG index has been validated as a reliable and accessible indicator of insulin resistance and has demonstrated good correlation with the hyperinsulinemic–euglycemic clamp technique. It has also been associated with an increased risk of metabolic syndrome, type 2 diabetes mellitus, and atherosclerotic cardiovascular disease. Higher TyG values reflect greater degrees of insulin resistance and adverse metabolic status [25].

In the present study, the TyG index was analyzed as a continuous variable to explore its relationship with cardiovascular and cognitive parameters.

#### 2.2.4. Cognitive and Functional Assessment

Cognitive function, including global cognition (attention, executive function, memory, language, and visuospatial functioning), was assessed using the MoCA. A score below 27 is indicative of mild cognitive impairment, and a score of less than 24 is indicative of moderate to severe impairment or severe cognitive impairment [17].

An MMSE score of 24–26 was considered indicative of probable cognitive impairment, whereas a score < 24 was considered indicative of probable severe cognitive impairment. These classifications were used for screening and analytical purposes only and do not represent a formal clinical diagnosis [15].

The ADL scale was used to assess basic functional abilities, including feeding, mobility, dressing, continence, and personal hygiene. Scores range from 0 to 10, with higher scores indicating better functional status [19].

The IADL scale looks at more complex measures of everyday living such as managing money, shopping and doing housework. The scores for this measure range from 0 to 8; therefore, higher scores indicate greater independence [18].

#### 2.2.5. Psychological Assessment

We assessed the patient for depressive symptomatology by employing the 15-item version of the Geriatric Depression Scale; a score between 0 and 4 indicates no depression, 5 to 8 indicates mild depression, 9 to 11 indicates moderate depression, and 12 to 15 indicates severe depression [21].

### 2.3. Definitions of Risk Factors

The traditional cardiovascular risk factors were identified based on criteria widely accepted in clinical practice and included the following variables: hypertension (defined as high blood pressure, i.e., the use of antihypertensive medications, a previous diagnosis of hypertension, or a measured blood pressure of 140 mm Hg or higher for systolic pressure or 90 mm Hg or higher for diastolic pressure); diabetes mellitus (defined as the use of antidiabetic medications, a prior diagnosis of diabetes, a fasting blood glucose level of 126 mg/dL or higher, or an HbA1c level of 6.5% or higher); dyslipidemia (defined as total cholesterol of 200 mg/dL or higher, LDL cholesterol of 130 mg/dL or higher, HDL cholesterol below 40 mg/dL for men and below 50 mg/dL for women, and triglycerides of 150 mg/dL or higher); smoking status (defined as a current or former user of tobacco products); body mass index (BMI) of 30 or higher; and family history of cardiovascular disease, defined as first-degree relatives with cardiovascular disease before the age of 55 for men and 65 for women. This definition is based on the current recommendations of the European Society of Cardiology [26] and the American Heart Association [27].

### 2.4. Statistical Analysis

Statistical analysis was performed using SPSS version 20.0 for Windows. Categorical percentage count data and continuous mean value and standard deviation data are presented in addition to the original text. Patients were compared by separating them into two groups: Group A represented all patients having CVRFs with CCS, whereas Group B represented patients having CVRFs but no CCS, the controls. For group A and group B, we used unpaired *t*-tests with a significance level of 0.05 to evaluate the blood tests (laboratory), HR, SBP, DBP, IMT, ABI, LV function measurements, memory, depression, as well as ADL/IADL tests. Comparison of the two groups for CVRFs with CCS vs. CVRFs without CCS will be calculated using an unpaired T-test. The association between categorical variables will also be assessed using the chi-square statistic and presented in the summary tables with the corresponding *p*-value. Spearman’s rank correlation coefficient was used to assess the correlation of key data. Bonnett and Wright’s method was used to estimate 95% confidence intervals (95% CIs) for the correlation coefficients.

After obtaining complete MMSE scores as a primary outcome measure, we used G * Power 3.1.9.2 software to calculate the achieved power for the MMSE for each of the two patient subgroups and found the following results: In comparing MMSE scores between groups A and B, we found an effect size of −1.33 corresponding to sample sizes. The G * Power 3.1.9.2 data indicates that the achieved power for this comparison was 99.99%. This result demonstrates that our sample size is sufficient to draw valid statistical inferences, as we achieved more than 80% power.

In order to determine prognostic factors for CD (MMSE scores between 24 and 27 and MMSE scores < 24) using age, SBP, DBP, HR, BMI and eGFR in model one and by using GDS-15 and ADL’s in model two with respect to CVRF’s and CCS patients (group A) and with respect to all study participants (group A and B) we employed the use of logistic regression employing forward selection with the Wald test in order to classify significant variables for which the odds ratios (OR) were presented along with 95% confidence intervals (CIs). To evaluate the quality of fit of the final regression models, we used Nagelkerke R-squared. Sensitivity, specificity, positive predictive value (PPV), and negative predictive value (NPV) were calculated for each independent variable of the last logistic regression model predicting risk of MMSE between 24 and 27 and the last logistic regression model predicting risk of MMSE < 24 (ADL and GDS-15) and an ROC curve and associated area under the ROC curve were also calculated.

## 3. Results

### 3.1. Study Population

A total of 264 participants, aged 70.72 (±9.72) years, with cardiovascular disease risk factors, were included. The subjects included 132 (50.0%) participants with CCS, and 132 (50.0%) without CCS. The average age of patients with CCS was 73.05 ± 8.62 years, compared with 68.39 ± 10.22 years in the control group (*p* < 0.001). The mean percentages of general compared to the control cohort had a very high prevalence of hypertension (89.8%), diabetes (50.4%), hyperlipidemia (55.3%), atrial fibrillation (48.9%), and chronic heart failure (84.1). Baseline characteristics between the groups were similar, and the other CVRFs did not differ significantly between the groups. Patients with CCS had a greater prevalence of carotid atherosclerosis < 50% than patients with alternative CCS. A more detailed breakdown of the characteristics can be found in Supplementary Table S1.

### 3.2. Comparative Analysis of Biologic Parameters in Both Groups of Patients (CVRFs With/Without CCS)

Compared to the group without CCS, both BMI and waist circumference were lower—but not statistically significant—for subjects with CCS. eGFR was also lower in CCS than in controls, but the difference did not reach statistical significance (57.45 ± 16.52 vs. 59.17 ± 18.03 mL/min). No significant differences were observed between groups for blood glucose, creatinine, urea, sodium, or potassium levels (Table 1).

Levels of LDL and HDL cholesterol in patients with CCS were significantly lower than those in control subjects (*p* < 0.05) based on the lipid profile. Total cholesterol, triglycerides, TyG index, and TG/HDL ratio were also lower in the CCS group; however, these differences were not statistically significant. INR was slightly lower for CCS patients, but not statistically significant compared with the control group. NT-proBNP was significantly higher among CCS patients (3100.29 ± 795.68 vs. 2825.58 ± 795.83 pg/mL, *p* = 0.005) (Supplementary Table S2).

### 3.3. Hemodynamic Parameters, IMT, and ABI

SBP was significantly higher in patients with CCS than in control subjects (133.18 ± 19.87 vs. 128.86 ± 21.10 mmHg), although the difference did not achieve statistical significance (*p* = 0.088). There was no significant difference in DBP between the two groups (79.13 ± 13.97 vs. 77.58 ± 15.06 mmHg; *p* = 0.386). However, HR was significantly lower in those with CCS than in the control group (72.77 ± 14.98 bpm vs. 76.73 ± 15.32 bpm; *p* = 0.034) (Table 2).

Patients diagnosed with CCS showed increased carotid artery thickness (IMT). The left side had an average of 0.75 mm (±0.26) compared to 0.68 mm for the controls (*p* = 0.024); the right side had an average thickness of 0.73 mm (±0.26) compared to controls who had an average of 0.66 mm (±0.28) (See Table 2). No significant differences in ankle-brachial index (ABI) values were observed between the two groups (Supplementary Table S3).

### 3.4. Echocardiographic Parameters

Left ventricular ejection fraction was significantly lower in patients with CCS compared to the control group (54.82 ± 6.80 vs. 57.81 ± 7.23%, *p* = 0.001). Global longitudinal strain values were also significantly affected in the CCS group (−16.96 ± 1.26 vs. −17.38 ± 1.02, *p* = 0.003) (Table 3).

Left ventricular end-diastolic diameter (LVEDD), left ventricular end-diastolic volume (LVEDV), and pulmonary systolic pressure (PSP) were higher in patients with CCS, while left ventricular end-diastolic diameter (LVEDD) and left ventricular end-diastolic volume (LVEDV) were lower compared with controls; however, these differences did not reach statistical significance. A complete overview of echocardiographic parameters is presented in Supplementary Table S4.

### 3.5. Neuropsychological Assessment

Neuropsychological assessment demonstrated significantly lower cognitive performance in patients with CCS compared with the control group, as reflected by lower MMSE and MoCA scores (*p* < 0.05). Functional status was also impaired, with lower ADL and IADL scores (*p* < 0.05), while GDS-15 scores were significantly higher, indicating a higher degree of depressive symptoms (*p* < 0.05) (Table 1). In the general cohort (n = 264), 181 patients (68.6%) had an MMSE score greater than 27, 34 (12.9%) had an MMSE score between 24 and 26, and 49 (18.6%) had an MMSE score less than 24.

Among the CCS patients (n = 132), 84 (63.6%) had an MMSE score > 27, 17 (12.9%) had an MMSE score 24–26, and 31 (23.5%) had an MMSE score < 24. In contrast, among the control group (n = 132), 97 (73.5%) had an MMSE score > 27, 17 (12.9%) had an MMSE score 24–26, and 18 (13.6%) had an MMSE score < 24.

**Table 1:** Neuropsychological assessment in patients with and without chronic coronary syndrome.

Parameter	CCS Group (n = 132)	Control Group (n = 132)	*p*-Value
MMSE	25.78 ± 4.72	27.11 ± 3.47	0.010
MoCA	23.41 ± 5.54	24.82 ± 4.23	0.021
ADL	9.11 ± 1.26	9.46 ± 1.00	0.012
IADL	6.50 ± 1.67	6.93 ± 1.53	0.030
GDS-15	7.24 ± 2.39	6.43 ± 2.14	0.004

Legend: MMSE: Mini-Mental State Examination Scale; MoCA: Montreal Cognitive Assessment Scale; ADL: Activities of Daily Living Score; IADL: Instrumental Activities of Daily Living Score; GDS-15: Geriatric Depression Scale 15 questions, *p*—statistical significance according to the unpaired *t*-test.

### 3.6. Correlation Analysis

In the general cohort (n = 264), age showed weak but statistically significant negative correlations with MMSE (r = −0.334, *p* < 0.001), MoCA (r = −0.133, *p* = 0.031), ADL (r = −0.210, *p* = 0.001) and IADL (r = −0.234, *p* < 0.001), as well as a weak positive correlation with GDS-15 (r = 0.181, *p* = 0.003). SBP demonstrated moderate negative correlations with MMSE (r = −0.449, *p* < 0.001) and MoCA (r = −0.461, *p* < 0.001), and weaker negative correlations with ADL (r = −0.225, *p* < 0.001) and IADL (r = −0.313, *p* < 0.001). A weak positive correlation was observed with GDS-15 (r = 0.359, *p* < 0.001). Similarly, DBP showed moderate negative correlations with MMSE (r = −0.433, *p* < 0.001) and MoCA (r = −0.447, *p* < 0.001), weak negative correlations with ADL (r = −0.269, *p* < 0.001) and IADL (r = −0.224, *p* < 0.001), and a weak positive correlation with GDS-15 (r = 0.406, *p* < 0.001). IMT was negatively correlated with cognitive and functional parameters. Left IMT showed weak to moderate negative correlations with MMSE (r = −0.253), MoCA (r = −0.385), ADL (r = −0.282), and IADL (r = −0.418), and a moderate positive correlation with GDS-15 (r = 0.541) (all *p* < 0.001). Similar findings were observed for right IMT (all *p* < 0.001). GDS-15 showed negative correlations with MMSE, MoCA, ADL, and IADL, with the strongest association observed for MoCA. In contrast, ADL and IADL were positively correlated with MMSE and MoCA (all *p* < 0.001). A detailed presentation of the correlation coefficients is presented in Supplementary Table S5.

Analysis of the full sample of patients (n = 264) revealed a weak but statistically significant correlation between FEVS and activities of daily living (r = 0.218, *p* < 0.001), as well as between instrumental activities of daily living (r = 0.291, *p* < 0.001) and a weak negative correlation between the GDS-15 and LVEF (r = −0.223, *p* < 0.001). The TyG index showed a very weak but statistically significant positive correlation with age (r = 0.138, *p* = 0.025). ABI (left) showed weak positive correlations with MoCA (r = 0.202, *p* = 0.001) and IADL (r = 0.173, *p* = 0.005), and a weak negative correlation with the GDS-15 (r = −0.243, *p* < 0.001). In patients with CCS (Group A), age showed a weak, negative correlation with the MMSE (r = −0.172, *p* = 0.048). Systolic and diastolic blood pressure were negatively correlated with both the MMSE and the MoCA at moderate levels *p* < 0.001) and showed weaker negative correlations with ADL and IADL *p* < 0.001. However, a weak-to-moderate positive correlation with the GDS-15 was evident among patients with CCS (*p* < 0.001).

Supplementary Table S6 illustrates that left IMT shows weak negative correlations with both the MMSE (r = −0.288, *p* = 0.001) and the MoCA (r = −0.307, *p* < 0.001), ADL (r = −0.259, *p* = 0.003), IADL (r = −0.323, *p* < 0.001), and a moderate positive correlation with the GDS-15 (r = 0.479, *p* < 0.001). Right IMT shows similar weak negative correlations with both the MMSE (r = −0.293, *p* = 0.001) and the MoCA (r = −0.214, *p* < 0.001), ADL (r = −0.248, *p* = 0.004), IADL (r = −0.308, *p* < 0.001), and a moderate positive correlation with the GDS-15 (r = −0.404, *p* < 0.001).

In patients with CCS and cardiovascular risk factors (Group A), LVEF showed a weak but statistically significant positive correlation with IADL (r = 0.222, *p* = 0.011). The ABI (left) was also weakly and positively correlated with MoCA (r = 0.201, *p* = 0.021) and IADL (r = 0.186, *p* = 0.033). In contrast, the ABI (left) showed a weak, but statistically significant, correlation with SBP (r = −0.293, *p* = 0.001), DBP (r = −0.258, *p* = 0.003), and GDS-15 (r = −0.264, *p* = 0.002).

### 3.7. Prognostic Factors for Cognitive Decline

Logistic regression analyses were performed to identify predictors of cognitive decline (CD; MMSE 24–27) and severe cognitive impairment (MMSE < 24) in the overall cohort (n = 264) and in patients with CCS (n = 132). Two models were constructed: Model 1 included demographic and clinical variables (age, SBP, DBP, HR, BMI, eGFR), while Model 2 evaluated functional and psychological parameters (ADL, GDS-15).

In Model 1, age, SBP, DBP, BMI, and eGFR were significant predictors of cognitive decline in the overall cohort, whereas age, DBP, BMI, and HR remained significant in CCS patients (Table 2). Age was the strongest determinant, with each additional year increasing the odds of CD by 10.3% in the overall cohort and 11.8% in CCS patients (*p* < 0.001). Increased SBP and DBP were also associated with increased risk, with DBP showing a more pronounced effect in CCS patients (+10.0% per mmHg). BMI independently increased the risk in both groups, while higher eGFR was associated with a reduced probability of CD in the overall cohort. The model showed moderate explanatory power (Nagelkerke R^2^ = 0.437–0.456).

**Table 2:** Multiple logistic regression analysis for the risk of MMSE between 24 and 27.

Patients with MMSE Between 24 and 27 n (%) *		OR (95% CI)	*p*-Value
264 CVRF patients with/without CCS * (group A and B)			
Age (years)	86 (32.58%)	1.103 (1.060; 1.147)	<0.001
SBP (mmHg)	86 (32.58%)	1.024 (1.004; 1.045)	0.020
DBP (mmH)	86 (32.58%)	1.042 (1.014; 1.072)	0.003
BMI (kg/m^2^)	86 (32.58%)	1.093 (1.030; 1.160)	0.003
eGFR (mL/min.)	86 (32.58%)	0.972 (0.953; 0.992)	0.006
132 patients with CCS and CVRFs (group A)			
Age (years)	49 (37.12%)	1.118 (1.052; 1.189)	<0.001
DBP (mmHg)	49 (37.12%)	1.100 (1.057; 1.145)	<0.001
HR (b/min.)	49 (37.12%)	1.032 (1.000; 1.065)	0.050
BMI (kg/m^2^)	49 (37.12%)	1.107 (1.011; 1.213)	0.028

Legend: n—number of patients, OR—odds ratio, 95% CI—95% confidence interval. * Percentages based on the total number of patients with CCS and CVRFs (134 patients) or the total number of patients with CVRFs with/without CCS included in the study.

For severe cognitive impairment (MMSE < 24), age, SBP, DBP, and BMI were consistent independent predictors in both groups (Table 3). Age remained the dominant factor, increasing severe cognitive impairment risk by 11.8% in the overall cohort and 13.4% in CCS patients (*p* < 0.001). Higher SBP and DBP contributed modest but significant increases in risk, while BMI had a stronger impact on CCS patients. Model performance was moderate (Nagelkerke R^2^ = 0.363–0.494).

**Table 3:** Multiple logistic regression analysis for risk of MMSE < 24. Prognostic factors for severe cognitive impairment, quantified by MMSE < 24 for age, SBP, DBP, and BMI in the entire sample.

Patients with MMSE < 24 (Severe Cognitive Impairment) n (%) *		OR (95% CI)	*p*-Value
264 CVRF patients with/without CCS (group A and B)			
Age (years)	55 (20.83%)	1.118 (1.067; 1.171)	<0.001
SBP (mmHg)	55 (20.83%)	1.022 (1.003; 1.042)	0.025
DBP (mmHg)	55 (20.83%)	1.045 (1.014; 1.076)	0.004
BMI (kg/m^2^)	55 (20.83%)	1.067 (1.000 1.138)	0.050
132 patients with CCS and CVRFs (group A)			
Age (years)	34 (25.76%)	1.134 (1.057; 1.216)	<0.001
SBP (mmHg)	34 (25.76%)	1.045 (1.012; 1.080)	0.008
DPB (mmHg)	34 (25.76%)	1.056 (1.007; 1.107)	0.023
BMI (kg/m^2^)	34 (25.76%)	1.106 (1.000; 1.223)	0.050

Legend: n—number of patients, OR—odds ratio, 95% CI—95% confidence interval. * Percentages based on the total number of patients with CCS and CVRFs (132 patients) or the total number of patients with CVRFs included in the study.

In Model 2, functional status and depressive symptoms were strongly associated with both cognitive decline and severe cognitive impairment (Table 4 and Table 5). Higher GDS-15 scores were associated with increased odds, whereas higher ADL scores were protective across both groups. In the overall cohort, each 1-point increase in GDS-15 increased the odds of CD by 41.0% (OR = 1.410, 95% CI: 1.215–1.636, *p* < 0.001), while each 1-point increase in ADL reduced the odds by 43.6% (OR = 0.564, 95% CI: 0.429–0.741, *p* < 0.001). Similar but slightly attenuated effects were observed in CCS patients (GDS-15: +32.1%, OR = 1.321; ADL: −43.5%, OR = 0.565; *p* < 0.05). Comparable associations were identified for severe cognitive impairment, with depression increasing risk and functional independence showing a protective effect.

**Table 4:** Multiple logistic regression analysis for the risk of MMSE between 24 and 27.

	Patients with MMSE Between 24 and 27 n (%) *	OR (95% CI)	*p*-Value
264 CVRF patients with/without CCS (group A and B)			
ADL	86 (32.58%)	0.564 (0.429; 0.741)	<0.001
GDS-15	86 (32.58%)	1.410 (1.215; 1.636)	<0.001
132 patients with CCS and CVRFs (group A)			
ADL	49 (37.12%)	0.565 (0.402; 0.794)	0.001
GDS-15	49 (37.12%)	1.321 (1. 094; 1.596)	0.004

Legend: n—number of patients, OR—odds ratio, 95% CI—95% confidence interval. * Percentages based on the total number of patients with CCS and CVRFs (132 patients) or the total number of patients with CVRFs with/without CCS included in the study.

**Table 5:** Multiple logistic regression analysis for risk of MMSE < 24. Prognostic factors for presenting severe cognitive impairment, quantified by MMSE < 24, for ADL and GDS-15 in all 248 CVRF patients with/without CCS (group A and B) or 132 patients with CCS and CVRFs (group A).

Patients with MMSE < 24 (Severe Cognitive Impairment) n (%) *		OR (95% CI)	*p*-Value
264 CVRF patients with/without CCS (group A and B)			
ADL	55 (20.83%)	0.558 (0.420; 0.741)	<0.001
GDS-15	55 (20.83%)	1.493 (1.241; 1.797)	<0.001
132 patients with CCS and CVRFs (group A)			
ADL	34 (25.75%)	0.561 (0.395; 0.796)	0.001
GDS-15	34 (25.75%)	1.371 (1.098; 1.713)	0.005

Legend: n—number of patients, OR—odds ratio, 95% CI—95% confidence interval. * Percentages based on the total number of patients with CCS and CVRFs (184 patients) or the total number of patients with CVRFs with/without CCS included in the study.

**Figure 1:**
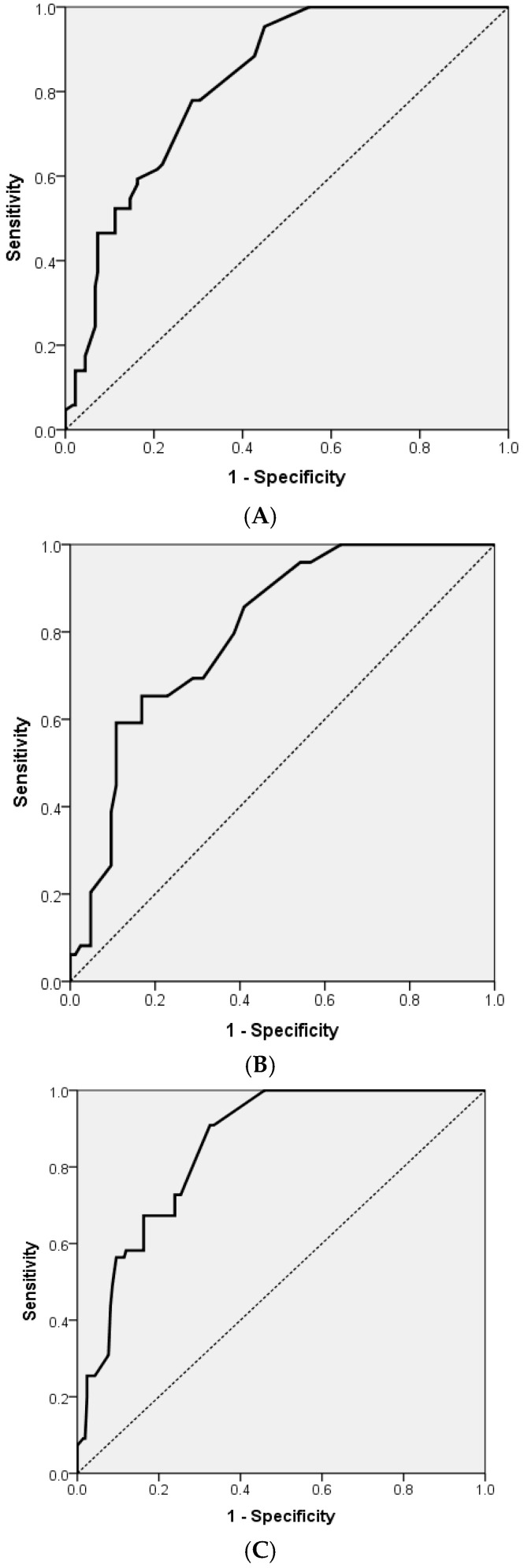

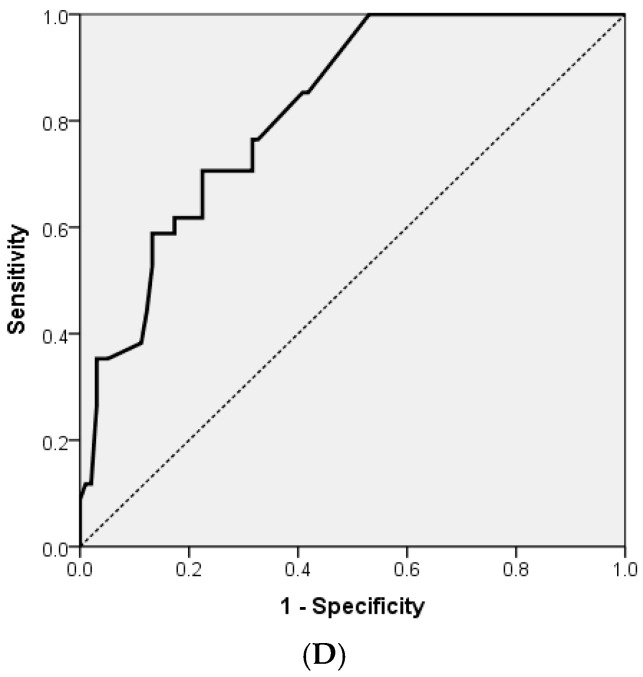
(**A**) ROC curve for multiple logistic regression model for the association of MMSE between 24 and 27 for patients with ADL score and GDS-15 score for the 264 CVRF patients with/without CCS (AUROC = 0.823, 95%CI 0.774; 0.872, *p* < 0.001). (**B**) ROC curve for multiple logistic regression model for the association of MMSE between 24 and 27 for patients with ADL score and GDS-15 score for the 132 patients with CCS and CVRFs (AUROC = 0.802, 95%CI 0.728; 0.876, *p* < 0.001). (**C**) ROC curve for multiple logistic regression model for the association of MMSE under 24 with ADL score and GDS-15 score for the 264 CVRF patients with/without CCS (AUROC = 0.853, 95%CI 0.807; 0.899, *p* < 0.001). (**D**) ROC curve for multiple logistic regression model for the association of MMSE under 24 with ADL score and GDS-15 score for the 132 patients with CCS and CVRFs (AUROC = 0.819, 95%CI 0.746; 0.893, *p* < 0.001).

Model discrimination was good, with AUROC values of 0.823 and 0.802 for CD and 0.853 and 0.819 for severe cognitive impairment in the overall cohort and CCS patients, respectively (Figure 1A–D). Classification accuracy ranged from 75% to 82%, with high specificity but moderate sensitivity.

Overall, these findings indicate that cognitive deterioration in patients with CCS is influenced by a combination of demographic, hemodynamic, metabolic, and psycho-social factors, with depression and functional decline emerging as key, potentially modifiable determinants.

## 4. Discussions

This study provides an in-depth assessment of the link between chronic coronary syndrome and cognitive decline by integrating neuropsychological, vascular, cardiac, functional, and psychological evaluations. The main findings of our study are that patients with CCS had significantly poorer cognitive performance, worse functional status, and more severe depressive symptoms than healthy controls with similar cardiovascular risk factors. Increased carotid intima-media thickness, hypertension, and impaired cardiac function were associated with poorer cognitive outcomes, thus supporting the dual vascular and cardiac contribution to cognitive decline. The emergence of depressive symptoms as among the strongest independent predictors of cognitive decline and severe cognitive impairment is another major finding, with preserved functional status playing a protective role. Our study is one of the few to evaluate CCS as a separate clinical entity, using a multidimensional approach that simultaneously includes cognitive, functional, vascular, echocardiographic, and psychological parameters. These results highlight the fact that cognitive impairments in CCS are multifactorial and, therefore, support the need for a comprehensive cardiovascular and cognitive assessment in this population.

### 4.1. Cognitive Impairment in Patients with Chronic Coronary Syndrome

Our main findings are that patients with CCS had considerably lower MMSE and MoCA scores than control subjects, despite the same burden of traditional cardiovascular risk factors. Unlike many studies looking at coronary artery disease as a broad whole, this study specifically looks at CCS and shows a strong link with cognitive and functional impairments.

These findings are consistent with previous studies linking coronary heart disease to cognitive decline [8,9]. Deckers et al. [12] reported that coronary heart disease was associated with a significantly increased risk of future cognitive decline and severe cognitive impairment. Meanwhile, Wolters et al. [11] have shown that both coronary heart disease and heart failure increase the risk of severe cognitive impairment. Also, according to their meta-analysis [11], the results showed that coronary heart disease was associated with a 26% increase in the risk of severe cognitive impairment in population-based cohorts.

These findings could be accounted for by several mechanisms. Chronic systemic inflammation, endothelial dysfunction, oxidative stress, and diffuse atherosclerosis may eventually result in cerebral hypoperfusion and neuronal injury [28]. Patients with CCS, however, are more likely to have multiple cardiovascular risk factors. Most of the patients have lower cognitive scores in our cohort. This would therefore support the idea that CCS should not just be considered as a cardiovascular disease but also as a marker for increased vulnerability to neurological conditions.

There are likely several ways in which CCS causes cognitive decline. In some cases, a lack of oxygen and blood flow to the brain, due to a number of factors, can damage brain cells and lead to their eventual death [6,8]. In addition, certain metabolic diseases, such as insulin resistance, have been shown to cause a decline in mental ability [29,30]. This data suggests that CCS and neurodegeneration have similar types of biological processes, which supports the rationale for a coordinated method for preventing and treating cognitive impairment associated with CCS.

### 4.2. Hemodynamic and Vascular Factors Associated with Cognitive Decline

A major finding in this study was the significant association between markers of vascular disease and cognitive performance. Patients with CCS had significantly higher values of carotid IMT. IMT had negative correlations with MMSE, MoCA, ADL, and IADL scores and was positively correlated with depressive symptoms. Our results suggest that subclinical atherosclerosis might be a key mechanistic link between CCS and cognitive decline.

Other investigators have made similar findings. Álvarez-Bueno et al. [31] showed that an increase in carotid IMT brings about high risks of cognitive impairment and severe cognitive impairment. Similarly, Gorelick et al. [32] stressed the primary role of vascular disease and cardiovascular risk factors in the pathogenesis of cognitive decline. The relationship between IMT and worse neuropsychological performance, as seen in our study, further supports the theory that chronic vascular injury results in cerebral hypoperfusion, microvascular damage, and progressive cognitive dysfunction.

Systolic and diastolic blood pressure were also significantly associated with poorer cognitive and functional outcomes. This finding is consistent with previous evidence showing that hypertension is related to cerebral small-vessel disease, white matter lesions, and accelerated cognitive decline [33]. Since blood pressure can be modified, aggressive management of cardiovascular risk factors could be an effective strategy for maintaining cognitive function in patients with CCS [34].

Hemodynamic and vascular factors are also major contributors to cognitive decline. In our study, higher systolic and diastolic blood pressure, along with increased carotid intima-media thickness, were all significantly correlated with poorer cognitive outcomes. IMT is an established marker of subclinical atherosclerosis and has been associated with cognitive impairment/severe cognitive impairment in many different studies [24]. Hypertension continues to be one of the most significant modifiable risk factors contributing to cognitive decline; data from randomized controlled trials and meta-analyses show that controlling blood pressure reduces the risk for severe cognitive impairment and mild cognitive impairment [35,36,37]. These results support the need for early identification and optimal management of vascular risk factors to prevent cognitive decline.

Cognitive decline in the elderly is the resultant effect of many factors and structural brain abnormalities, such as silent cerebral infarctions, lacunar infarctions, white matter disease, cerebral atrophy, and other neurodegenerative or vascular changes, which may have a substantial effect on cognitive performance. Brain MRI and other standardized neuroimaging studies were not performed in all participants, and hence, the causes of cognitive decline could not be fully determined [38].

All in all, these findings underscore the importance of early identification and optimal management of vascular risk factors in CCS patients. They also further support the suggestion that routine assessment of blood pressure and markers of subclinical atherosclerosis can help in identifying those at higher risk of cognitive decline prior to the development of manifest severe cognitive impairment.

### 4.3. Cardiac Function and the Heart–Brain Axis

Impaired cardiac function was found in patients with CCS. Left ventricular ejection fraction was significantly lower, and global longitudinal strain values were less favorable in CCS patients compared to controls. These results further support the concept of the heart-brain axis, which is still considered new, and propose that even relatively modest cardiac dysfunction may contribute to cognitive impairment.

This relationship has been described in previous studies. Jefferson et al. [39] found that diminished cardiac output was associated with smaller brain volumes and worse cognitive performance, even in the absence of clinical heart failure. In a similar line of thought, Iadecola and Gottesman suggested that cardiovascular dysfunction might be a potentially modifiable contributor to neurodegeneration and cognitive decline [40].

Reduced cerebral perfusion as a result of impaired cardiac function may therefore contribute to chronic ischemic injury and neuronal dysfunction. Furthermore, neurohormonal activation, inflammation, and endothelial dysfunction may amplify the adverse effects of cardiovascular disease on the brain [39,40]. These results support the hypothesis that both vascular and cardiac abnormalities contribute to cognitive impairments in patients with CCS.

### 4.4. Depression, Functional Status, and Cognitive Outcomes

Another key finding of our study is the close link between depressive symptoms, functional status, and cognitive impairments. Higher GDS-15 scores were associated with increased odds of both cognitive decline and severe cognitive impairment, whereas higher ADL scores had a protective effect. Notably, depression emerged as one of the strongest independent predictors of adverse cognitive outcomes in our cohort, highlighting the importance of psychological factors in the development and progression of cognitive impairment among patients with CCS. These findings are consistent with previous research indicating that depression may act both as a risk factor and as a prodromal manifestation of cognitive impairment [41]. Functional decline, reflected by reduced ADL and IADL scores, has also been associated with progression from mild cognitive impairment to severe cognitive impairment [18,19]. The interaction between depression, reduced functional capacity, and cognitive decline suggests a complex and bidirectional relationship that may accelerate disease progression.

Although blood-based biomarkers for Alzheimer’s disease were not evaluated in the present study, recent advances suggest that markers such as plasma p-tau, Aβ42/40 ratio, neurofilament light chain, and glial fibrillary acidic protein may improve the identification of individuals at increased risk of progressive cognitive decline and severe cognitive impairment [42]. Future studies integrating cardiovascular assessment with neuroimaging and biomarker evaluation may help clarify the mechanisms underlying cognitive impairment in patients with CCS.

So, it is possible that the connections between heart problems and memory problems could actually be caused by a combination of problems with the heart and other illnesses like Alzheimer’s that already existed before either heart problems or memory problems started showing up. Studies have found links between different kinds of blood vessel problems (such as high blood pressure) and a risk of developing Alzheimer’s disease through the formation of toxic protein clumps in the brain [43,44]. Evidence from hospitals caring for patients with heart attacks shows that quick medical care that improves blood vessel function can have either a positive or a negative impact on brain function [45].

Integrating biomarker assessment, along with cardiovascular and neuropsychological evaluations from a clinical standpoint, could enable earlier identification of high-risk patients and enable clinicians to develop more personalized therapeutic strategies. Improving treatment adherence, increasing participation in cardiac rehabilitation, and slowing the progression of cognitive impairment and severe cognitive impairment are expected outcomes of this approach [42,46].

### 4.5. Clinical Implications

The findings highlight the importance of incorporating cognitive and functional assessment into routine care for patients with CCS. Although current guidelines primarily focus on the management of cardiovascular risk factors, cognitive evaluation is not routinely performed in cardiology practice [1,47]. Early detection of cognitive impairment may improve treatment adherence, enhance participation in cardiac rehabilitation, and optimize long-term outcomes. Multidomain interventions, including lifestyle modification, physical activity, cognitive training, and optimal control of cardiovascular risk factors, have shown promising results in preventing cognitive decline [48,49].

Our results indicate that cognitive screening should be performed in all CCS patients, more so in old patients and those showing symptoms of vascular disease, depression, or functional decline.

The identification of modifiable predictors, including hypertension, depressive symptoms, and reduced functional capacity, highlights potential opportunities for intervention. A multidisciplinary approach involving cardiologists, neurologists, psychiatrists, geriatricians, and primary care physicians may facilitate earlier recognition of cognitive dysfunction and improve both cardiovascular and neurological outcomes.

### 4.6. Study Limitations and Future Directions

It is important to clarify the substantial number of study limitations. To begin, the study used a relatively small, single-site sample, which limits the generalizability of these results to broader populations. Secondly, the study design prevents identification of cause and effect between chronic coronary syndrome and cognitive decline, or the determination of the temporal order of cognitive decline. Thirdly, baseline differences in group characteristics such as age distribution may have affected the association regardless of the performed statistical adjustment for these variables.

Also, many possible factors like socioeconomic status, medication, treatment for depressive symptoms, and sleep disorders or their level of education, can affect them cognitively, but were not measured in this analysis. Cognitive function was also evaluated using screening instruments (MMSE, MoCA), which do not identify subtle domain-specific deficits across all cognitive domains. Similarly, measurements of biomarkers and hemodynamic parameters may vary depending on laboratory methodology and clinical circumstances, potentially affecting reproducibility. Unfortunately, detailed angiographic characteristics (e.g., number of diseased vessels, SYNTAX score, lesion location, or completeness of revascularization) were not systematically available for all participants and therefore could not be included in the analysis.

Several limitations should be considered when interpreting the findings of this study. Despite efforts to recruit participants with broadly comparable age distributions, the CCS group was significantly older than the control group. Given that age is a well-established risk factor for cognitive decline and severe cognitive impairment, residual confounding cannot be completely excluded. Although age was included as a covariate in the multivariable logistic regression analyses, part of the observed differences in cognitive performance may still be attributable to age-related effects. Second, all participants were recruited from individuals reporting progressive memory impairment or neurocognitive symptoms. Consequently, the study population was enriched with subjects already at increased risk of cognitive dysfunction, potentially introducing selection bias and limiting the ability to determine the independent contribution of CCS to cognitive decline. Therefore, the findings cannot be generalized to asymptomatic individuals or the broader CCS population. Future studies using individual age matching, propensity score methods, larger cohorts, and the inclusion of participants without subjective cognitive complaints are needed to further clarify the relationship between CCS and cognitive impairment. Another limitation is the lack of detailed information regarding medication use. Pharmacological therapies commonly prescribed in patients with CCS, including statins, beta-blockers, ACE inhibitors/ARBs, and antidepressants, may influence cognitive function, mood, functional status, and cardiovascular outcomes. Because these data were not systematically collected, their potential confounding effects could not be assessed or adjusted for in the analyses.

The final limitation on evaluating the changes from mild cognitive impairment to severe cognitive impairment and long-term clinical outcomes is the absence of longitudinal follow-up data. The validation of these findings and the evaluation of long-term clinical outcomes will require large-scale, multicenter, and prospective studies that use advanced imaging and blood biomarker measurement technologies.

However, the study also has very important strengths. We evaluated CCS as a distinct clinical entity, which has not been performed in many other studies, and combined detailed neuropsychological testing with vascular, echocardiographic, functional, and psychological assessments. In this way, this multidimensional approach enabled us to identify several potentially modifiable factors associated with cognitive decline.

More extended future prospective multicenter studies with advanced neuroimaging and circulating biomarkers along with longer follow-up periods are required to confirm the above findings and clarify the causal pathways linking CCS and cognitive decline. Interventional studies are also needed to check whether optimization of cardiovascular risk factors, treatment of depression, and targeted rehabilitation strategies can reduce the risk of cognitive deterioration in this population.

## 5. Conclusions

The study highlights the need for cognitive and functional screening in patients with CCS for early identification of patients at high risk of CD, severe cognitive impairment, and disability. Patients with CCS have significantly lower cognitive scores (both MMSE and MoCA), respectively, functional autonomy (assessed by ADL and IADL scales), respectively, depression elements (assessed by GDS 15 scales) compared to patients with CVRFs without CCS. Also, patients with CCS had significantly higher levels of cardiovascular and metabolic biomarkers (carotid IMT and NT-pro-BNP), and LVEF was significantly lower than in patients with CVRFs without CCS. Increasing age, increasing systolic and diastolic blood pressure, and increasing markers of subclinical atherosclerosis are statistically significantly correlated with decreasing cognitive scores, respectively, decreasing functional autonomy, and increasing elements of depression. Altered vascular and metabolic risk markers may lead to decreased independence in daily activities among patients with CCS; thus, assessment and monitoring of functional and cognitive scores are particularly important for appropriate management. Integrating cognitive assessments (MMSE, MoCA), functional elements (ADL/IADL), and depression (GDS 15) in the management of patients with CCS may guide early therapeutic interventions and prevent disability progression. Future research should focus on longitudinal, biomarker-based, and interventional studies to elucidate the heart–brain mechanisms underlying cognitive decline in chronic coronary syndrome and to develop effective prevention strategies.

## Data Availability

The data supporting the reported results are available from the first author of this manuscript, Marius Militaru, upon request.

## Supplementary Materials

The following supporting information can be downloaded at: https://www.mdpi.com/article/10.3390/medicina62071239/s1, Table S1. Baseline demographic characteristics, cardiovascular risk factors, and comorbidities in patients with and without chronic coronary syndrome. Table S2. Laboratory parameters in patients with and without chronic coronary syndrome. Table S3. Hemodynamic parameters, carotid intima–media thickness, and ankle–brachial index in patients with and without chronic coronary syndrome. Table S4. Echocardiographic parameters of left ventricular systolic and diastolic function in patients with and without chronic coronary syndrome. Table S5. Correlation between age, SBP, DBP, IMT left/right and MMSE, MoCA, ADL, IADL, and GDS-15 for all 264 CVRFs with/without CCS from the study. Table S6. Correlation between age, SBP, DBP, IMT (left/right), and MMSE, MoCA, ADL, IADL, and GDS-15 in 132 patients with CVRFs and CCS (group A).
