# Supplementary material for: Cognitive Decline in Chronic Coronary Syndrome: Associations with Vascular, Cardiac, and Neuropsychological Parameters
Source: Medicina (Kaunas). 2026 Jun 26;62(7):1239. doi: 10.3390/medicina62071239 (PMC13414391; doi:10.3390/medicina62071239)
Supplement: Supplementary file 1 [file medicina-62-01239-s001.zip › Supplementary Table S1.pdf]

Supplementary Table S1. Baseline demographic characteristics, cardiovascular risk factors, and comorbidities in patients with and without chronic coronary syndrome

Variable	CCS Group (n = 132)	Control Group (n = 132)	p-value
**Age, years**	73.05 ± 8.62	68.39 ± 10.22	<0.001*
**Sex, n (%)**			
Male	65 (49.2)	57 (43.2)	0.323
Female	67 (50.8)	75 (56.8)	0.323
**Hypertension, n (%)**	116 (87.9)	121 (91.7)	0.310
**Type 2 diabetes mellitus, n (%)**	73 (55.3)	60 (45.5)	0.110
**Smoking, n (%)**	44 (33.3)	38 (28.8)	0.425
**Atrial fibrillation, n (%)**	69 (52.3)	60 (45.5)	0.268
└ Paroxysmal	31 (23.5)	27 (20.5)	0.552
└ Persistent	15 (11.4)	14 (10.6)	0.844
└ Permanent	23 (17.4)	19 (14.4)	0.501
**Hyperlipidemia, n (%)**	70 (53.0)	76 (57.6)	0.458
**Obesity, n (%)**	23 (17.4)	34 (25.8)	0.100
└ Grade I	14 (10.6)	19 (14.4)	0.352
└ Grade II	8 (6.1)	13 (9.8)	0.255
└ Grade III	1 (0.8)	1 (0.8)	>0.999
**Chronic kidney disease, n (%)**	86 (65.2)	72 (54.5)	0.079
└ Stage I	26 (19.7)	13 (9.8)	0.024
└ Stage II	24 (18.2)	25 (18.9)	0.874
└ Stage III	29 (22.0)	28 (21.2)	0.881
└ Stage IV	7 (5.3)	6 (4.5)	0.776
**Peripheral arterial disease, n (%)**	14 (10.6)	6 (4.5)	0.063
**Chronic heart failure, n (%)**	114 (86.4)	108 (81.8)	0.313
└ NYHA I	10 (7.6)	12 (9.1)	0.656
└ NYHA II	70 (53.0)	58 (43.9)	0.139
└ NYHA III	27 (20.5)	31 (23.5)	0.552
└ NYHA IV	7 (5.3)	7 (5.3)	>0.999
**Carotid atherosclerosis <50%, n (%)**	57 (43.2)	40 (30.3)	0.030*
**Carotid stenosis >50%, n (%)**	10 (7.6)	7 (5.3)	0.452
**Lacunar stroke, n (%)**	21 (15.9)	13 (9.8)	0.142
**Minor stroke, n (%)**	14 (10.6)	14 (10.6)	>0.999

Abbreviations: CCS, chronic coronary syndrome; AF, atrial fibrillation; CKD, chronic kidney disease; CHF, chronic heart failure; NYHA, New York Heart Association; T2DM, type 2 diabetes mellitus.
**Statistically significant ($p < 0.05$).*
