# Supplementary material for: Cognitive Decline in Chronic Coronary Syndrome: Associations with Vascular, Cardiac, and Neuropsychological Parameters
Source: Medicina (Kaunas). 2026 Jun 26;62(7):1239. doi: 10.3390/medicina62071239 (PMC13414391; doi:10.3390/medicina62071239)
Supplement: Supplementary file 1 [file medicina-62-01239-s001.zip › Supplementary Table S2.pdf]

Supplementary Table S2. Laboratory parameters in patients with and without chronic coronary syndrome (CCS)

Parameter	CCS Group (n = 132)	Control Group (n = 132)	p-value
BMI (kg/m²)	28.56 ± 5.82	27.44 ± 5.18	0.101
Waist circumference (cm)	88.68 ± 6.61	89.57 ± 7.88	0.323
Total cholesterol (mg/dL)	190.46 ± 57.94	178.08 ± 64.28	0.101
LDL-C (mg/dL)	143.86 ± 54.55	126.02 ± 55.41	0.009
HDL-C (mg/dL)	49.84 ± 19.37	45.28 ± 14.88	0.033
Triglycerides (mg/dL)	135.83 ± 57.85	126.44 ± 55.78	0.181
TG/HDL ratio	3.14 ± 1.97	3.11 ± 1.85	0.909
Uric acid (mg/dL)	6.21 ± 1.56	6.32 ± 1.48	0.567
Fasting glucose (mg/dL)	109.11 ± 21.81	109.86 ± 29.74	0.814
Creatinine (mg/dL)	1.00 ± 0.25	0.97 ± 0.21	0.202
Urea (mg/dL)	41.71 ± 19.64	41.54 ± 15.58	0.938
eGFR (mL/min/1.73 m²)	57.45 ± 16.52	59.17 ± 18.03	0.421
TyG index	4.73 ± 0.27	4.71 ± 0.24	0.465
CK-MB (U/L)	17.81 ± 9.55	16.51 ± 9.43	0.267
Sodium (mmol/L)	140.58 ± 3.21	140.64 ± 2.59	0.872
Potassium (mmol/L)	4.10 ± 0.47	4.04 ± 0.44	0.270
INR	1.88 ± 0.75	1.78 ± 0.77	0.310
**NT-proBNP (pg/mL)**	**3100.29 ± 795.68**	**2825.58 ± 795.83**	**0.005**

Abbreviations: BMI, body mass index; LDL-C, low-density lipoprotein cholesterol; HDL-C, high-density lipoprotein cholesterol; TG, triglycerides; eGFR, estimated glomerular filtration rate; TyG, triglyceride-glucose index; CK-MB, creatine kinase myocardial band; INR, international normalized ratio; NT-proBNP, N-terminal pro–B-type natriuretic peptide.

Data are presented as mean ± standard deviation. p-values were calculated using the unpaired t-test. Statistically significant differences ($p < 0.05$) are shown in bold.
