# Supplementary material for: Cognitive Decline in Chronic Coronary Syndrome: Associations with Vascular, Cardiac, and Neuropsychological Parameters
Source: Medicina (Kaunas). 2026 Jun 26;62(7):1239. doi: 10.3390/medicina62071239 (PMC13414391; doi:10.3390/medicina62071239)
Supplement: Supplementary file 1 [file medicina-62-01239-s001.zip › Supplementary Table S3.pdf]

Supplementary Table S3. Hemodynamic parameters, carotid intima–media thickness, and ankle–brachial index in patients with and without chronic coronary syndrome (CCS)

Parameter	CCS Group (n = 132)	Control Group (n = 132)	p-value
SBP (mmHg)	133.18 ± 19.87	128.86 ± 21.10	0.088
DBP (mmHg)	79.13 ± 13.97	77.58 ± 15.06	0.386
**HR (beats/min)**	**72.77 ± 14.98**	**76.73 ± 15.32**	**0.034**
**Left IMT (mm)**	**0.75 ± 0.26**	**0.68 ± 0.24**	**0.024**
**Right IMT (mm)**	**0.73 ± 0.26**	**0.66 ± 0.28**	**0.035**
Left ABI	1.07 ± 0.11	1.08 ± 0.10	0.751
Right ABI	1.07 ± 0.17	1.08 ± 0.10	0.689

Abbreviations: SBP, systolic blood pressure; DBP, diastolic blood pressure; HR, heart rate; IMT, carotid intima–media thickness; ABI, ankle–brachial index.

Data are presented as mean ± standard deviation. p-values were calculated using the unpaired t-test. Statistically significant differences ($p < 0.05$) are shown in bold.
