# Supplementary material for: Cognitive Decline in Chronic Coronary Syndrome: Associations with Vascular, Cardiac, and Neuropsychological Parameters
Source: Medicina (Kaunas). 2026 Jun 26;62(7):1239. doi: 10.3390/medicina62071239 (PMC13414391; doi:10.3390/medicina62071239)
Supplement: Supplementary file 1 [file medicina-62-01239-s001.zip › Supplementary Table S4.pdf]

Supplementary Table S4. Echocardiographic parameters of left ventricular systolic and diastolic function in patients with and without chronic coronary syndrome (CCS)

Parameter	CCS Group (n = 132)	Control Group (n = 132)	p-value
IVS (mm)	11.98 ± 1.95	11.96 ± 2.29	0.931
LA (mm)	41.38 ± 6.24	41.15 ± 7.04	0.782
LVPW (mm)	12.36 ± 2.39	12.12 ± 2.58	0.427
LVESD (mm)	24.23 ± 6.53	24.08 ± 6.73	0.853
LVEDD (mm)	46.09 ± 5.94	45.40 ± 4.88	0.299
LVESV (mL)	36.78 ± 14.99	35.95 ± 12.41	0.626
LVEDV (mL)	75.43 ± 16.92	73.64 ± 17.42	0.397
**LVEF (%)**	**54.82 ± 6.80**	**57.81 ± 7.23**	**0.001**
E (m/s)	0.68 ± 0.17	0.71 ± 0.17	0.159
A (m/s)	0.71 ± 0.18	0.72 ± 0.17	0.660
E/A ratio	0.68 ± 0.16	0.71 ± 0.16	0.174
Tricuspid Vmax (m/s)	2.37 ± 0.51	2.33 ± 0.48	0.473
sPAP (mmHg)	38.72 ± 12.08	37.99 ± 11.78	0.617
**GLS (%)**	**−16.96 ± 1.26**	**−17.38 ± 1.02**	**0.003**
E′/A′ ratio	1.47 ± 1.01	1.52 ± 1.10	0.686
S′ (cm/s)	11.77 ± 3.15	12.47 ± 3.02	0.068
MAPSE (mm)	12.59 ± 0.74	12.68 ± 0.82	0.342

Abbreviations: IVS, interventricular septal thickness; LA, left atrial diameter; LVPW, left ventricular posterior wall thickness; LVESD, left ventricular end-systolic diameter; LVEDD, left ventricular end-diastolic diameter; LVESV, left ventricular end-systolic volume; LVEDV, left ventricular end-diastolic volume; LVEF, left ventricular ejection fraction; E, peak early transmitral diastolic flow velocity; A, peak late transmitral diastolic flow velocity; E/A, ratio of early-to-late transmitral flow velocity; sPAP, systolic pulmonary artery pressure; GLS, global longitudinal strain; E′, peak early mitral annular diastolic velocity; A′, peak late mitral annular diastolic velocity; S′, peak mitral annular systolic velocity; MAPSE, mitral annular plane systolic excursion.

Data are presented as mean ± standard deviation. p-values were calculated using the unpaired t-test. Statistically significant differences ($p < 0.05$) are shown in bold.
