# Supplementary material for: Cognitive Decline in Chronic Coronary Syndrome: Associations with Vascular, Cardiac, and Neuropsychological Parameters
Source: Medicina (Kaunas). 2026 Jun 26;62(7):1239. doi: 10.3390/medicina62071239 (PMC13414391; doi:10.3390/medicina62071239)
Supplement: Supplementary file 1 [file medicina-62-01239-s001.zip › Supplementary Table S5.pdf]

Supplementary Table S5. Correlation between age, SBP, DBP, IMT left/right and MMSE, MoCA, ADL, IADL, and GDS-15 for all 264 CVRFs with/without CCS from the study.

Parameter	MMSE	MoCA	ADL	IADL	GDS-15
**Age**					
r	−0.334	−0.133	−0.210	−0.234	0.181
95%CI	−0.440; −0.219	−0.250; −0.012	−0.324; −0.090	−0.346; −0.115	0.061; 0.296
p	<0.001	0.031	0.001	<0.001	0.003
**SBP (mmHg)**					
r	- 0.449	-0.461	-0.225	-0.313	0.359
95%CI	−0545.; -0.342	−0.555; -0.355	-0.338; -0.106	-0.420; -0.197	0.245; 0.463
p	<0.001	<0.001	<0.001	<0.001	<0.001
**DPB (mmHg)**					
r	−0.433	−0.447	−0.269	−0.224	0.406
95%CI	−0.530; −0.325	−0.543; −0.340	−0.379; −0.151	−0.337; −0.105	0.296; 0.506
p	<0.001	<0.001	<0.001	<0.001	<0.001
**IMT left (mm)**					
r	−0.253	−0.385	−0.282	−0.418	0.541
95%CI	−0.364; −0.135	−0.487; −0.273	−0.391; −0.165	−0.517; −0.308	0.443; 0.626
p	<0.001	<0.001	<0.001	<0.001	<0.001
**IMT right (mm)**					
r	-0.287	-0.307	-0.292	-0.341	0.455
95%CI	−0.396; −0.170	−0.415; −0.191	−0.401; −0.175	−0.446; −0.226	−0.348; −0.550
p	<0.001	<0.001	<0.001	<0.001	<0.001
**GDS-15**					
r	−0.473	−0.607	−0.449	-0.569	
95%CI	−0.566; −0.368	−0.684; −0.517	−0.545; −0.342	-0.651; -0.474	
p	<0.001	<0.001	<0.001	<0.001	
**IADL**					
r	0.385	0.431	0.584		
95%CI	0.273; 0.487	0.322; 0.528	0.491; 0.664		
p	<0.001	<0.001	<0.001		
**ADL**					
r	0.455	0.408			
95%CI	0.348; 0.550	0.298; 0.508			
p	<0.001	<0.001			
**MoCA**					
r	0.657				
95%CI	0.574; 0.727				
p	<0.001				

Legend: MMSE—Mini-Mental State Examination Scale; MoCA—Montreal Cognitive Assessment Scale; ADL—Activities of Daily Living Score, IADL—Instrumental Activities of Daily Living Score; GDS-15—Geriatric Depression Scale 15 questions; SBP—systolic blood pressure; DBP—diastolic blood pressure; IMT - intima–media thickness; r—Spearman's correlation coefficient, 95% CI = 95% confidence interval estimated using Bonnett and Wright's method.
