# Supplementary material for: Cognitive Decline in Chronic Coronary Syndrome: Associations with Vascular, Cardiac, and Neuropsychological Parameters
Source: Medicina (Kaunas). 2026 Jun 26;62(7):1239. doi: 10.3390/medicina62071239 (PMC13414391; doi:10.3390/medicina62071239)
Supplement: Supplementary file 1 [file medicina-62-01239-s001.zip › Supplementary Table S6.pdf]

Supplementary Table S6. Correlation between age, SBP, DBP, IMT (left/right), and MMSE, MoCA, ADL, IADL, and GDS-15 in 132 patients with CVRFs and CCS (group A).

Parameter	MMSE	MoCA	ADL	IADL	GDS-15
**Age**					
r	−0.172	−0.032	−0.004	0.046	-0.031
95%CI	−0.334; −0.0001	−0.202; −0.140	−0.175; −0.167	-0.126; -0.215	-0.201; 0.141
p	0.048	0.719	0.961	0.603	0.723
**SBP (mmHg)**					
r	- 0.475	-0.526	-0.250	-0.253	0.327
95%CI	−0.603; -0.323	−0.646; -0.380	-0.406; -0.080	-0.409; -0.830	0.161; 0.475
p	<0.001	<0.001	<0.001	0.003	<0.001
**DPB (mmHg)**					
r	−0.517	−0.542	−0.276	−0.223	0.440
95%CI	−0.639; −0.370	−0.659; −0.399	−0.429; −0.107	−0.381; −0.052	0.284; 0.574
p	<0.001	<0.001	0.001	0.010	<0.001
**IMT left (mm)**					
r	−0.288	−0.307	−0.259	−0.323	0.479
95%CI	−0.440; −0.120	−0.457; −0.140	−0.414; −0.089	−0.472; −0.157	0.327; 0.607
p	0.001	<0.001	0.003	<0.001	<0.001
**IMT right (mm)**					
r	-0.293	-0.214	-0.248	-0.308	0.404
95%CI	−0.445; −0.125	−0.373; −0.043	−0.404; −0.078	−0.458; −0.141	0.244; 0.543
p	0.001	0.014	0.004	<0.001	<0.001
**GDS-15**					
r	−0.475	−0.625	−0.436	-0.467	
95%CI	−0.603; −0.323	−0.727; −0.496	−0.571; −0.279	-0.597; -0.314	
p	<0.001	<0.001	<0.001	<0.001	
**IADL**					
R	0.326	0.344	0.562		
95%CI	0.160; 0.474	0.179; 0.490	0.422; 0.676		
p	<0.001	<0.001	<0.001		
**ADL**					
r	0.471	0.422			
95%CI	0.318; 0.600	0.264; 0.558			
p	<0.001	<0.001			
**MoCA**					
r	0.781				
95%CI	0.691; 0.847				
p	<0.001				

Legend: MMSE—Mini-Mental State Examination Scale; MoCA—Montreal Cognitive Assessment Scale; ADL—Activities of Daily Living Score, IADL—Instrumental Activities of Daily Living Score; GDS-15—Geriatric Depression Scale 15 questions; SBP—systolic blood pressure; DBP—diastolic blood pressure; IMT - intima–media thickness ; r—Spearman's correlation coefficient, 95% CI = 95% confidence interval estimated using Bonnett and Wright's method.
